# Downregulated miR-181a alleviates H_2_O_2_-induced oxidative stress and cellular senescence by targeting PDIA6 in human foreskin fibroblasts^☆^

**DOI:** 10.1016/j.abd.2021.12.007

**Published:** 2022-10-14

**Authors:** Yan Huang, Huimin Yan, Yanqing Yang, Jinfei Zhou, Qijun Xu, Hu Meng

**Affiliations:** a Wuhan Third Hospital, Department of Plastic Surgery, Hubei, China; b Xiangyang Yilaimei Medical Beauty Clinic, Department of Aesthetic Dermatology, Hubei, China

**Keywords:** Cellular senescence, Oxidative stress, Protein disulfide-isomerases

## Abstract

**Background:**

Oxidative stress is strongly associated with cellular senescence. Numerous studies have indicated that microRNAs (miRNAs) play a critical part in cellular senescence. MiR-181a was reported to induce cellular senescence, however, the potential mechanism of miR-181a in hydrogen peroxide (H_2_O_2_)-induced cellular senescence remains obscure.

**Objective:**

The aim of this study is to investigate the role and regulatory mechanism of miR-181a in H_2_O_2_-induced cellular senescence.

**Methods:**

Human foreskin fibroblasts (HFF) transfected with miR-181a inhibitor/miR-NC with or without H_2_O_2_ treatment were divided into four groups: control + miR-NC/miR-181a inhibitor, H_2_O_2_ + miR-NC/miR-181a inhibitor. CCK-8 assay was utilized to evaluate the viability of HFF. RT-qPCR was used to measure the expression of miR-181a and its target genes. Protein levels of protein disulfide isomerase family A member 6 (PDIA6) and senescence markers were assessed by western blotting. Senescence-associated β-galactosidase (SA-β-gal) staining was applied for detecting SA-β-gal activity. The activities of SOD, GPx, and CAT were detected by corresponding assay kits. The binding relation between PDIA6 and miR-181a was identified by luciferase reporter assay.

**Results:**

MiR-181a inhibition suppressed H_2_O_2_-induced oxidative stress and cellular senescence in HFF. PDIA6 was targeted by miR-181a and lowly expressed in H_2_O_2_-treated HFF. Knocking down PDIA6 reversed miR-181a inhibition-mediated suppressive impact on H_2_O_2_-induced oxidative stress and cellular senescence in HFF.

**Study limitations:**

Signaling pathways that might be mediated by miR-181a/PDIA6 axis were not investigated.

**Conclusion:**

Downregulated miR-181a attenuates H_2_O_2_-induced oxidative stress and cellular senescence in HFF by targeting PDIA6.

## Introduction

Cellular senescence is a process comprised of irreversible growth arrest which is regarded as one of the hallmarks of aging.^1^ Cellular senescence in the skin leads to skin aging which can be induced by both extrinsic and intrinsic factors.^2^, ^3^ Senescent cells can be detected with many hallmarks, such as enhanced activity of senescence-associated β-galactosidase (SA-β-gal), elevated levels of tumor suppressor p53, cyclin-dependent kinase (CDK) inhibitor p21 and senescence marker protein 30 (SMP30).^4^, ^5^ Numerous studies have indicated that oxidative stress contributes to skin aging and dermal damage.^6^ Oxidative stress is a physiological process resulting from reactive oxygen (ROS) or nitrogen species, including hydrogen peroxide (H_2_O_2_) and superoxide anion (O^2−^).^7^ The skin has an antioxidative defense system, including the enzymatic antioxidants like superoxide dismutase (SOD), glutathione peroxidase (GPx), and catalase (CAT), which helps to eliminate excessive ROS.^8^ SOD converts O^2−^ into H_2_O_2,_ while GPx and CAT convert H_2_O_2_ into water.^9^, ^10^

It has been elucidated that microRNAs (miRNAs), endogenous noncoding RNAs of 18–24 nucleotides, are implicated in a variety of biological processes.^11^, ^12^ MiRNAs can bind to messenger RNA (mRNA) 3’ untranslated regions (3’UTRs) and regulate gene expression post-transcriptionally.^13^ Multiple miRNAs have been indicated to play an important role in cellular senescence in the skin. For example, miR-217 facilitates senescence in human skin fibroblasts by binding to DNMT1.^14^ MiR-20a-3p is overexpressed in senescent fibroblasts and leads to cellular senescence by targeting HAS2.^15^ Importantly, miR-181a was also demonstrated to be involved in cellular senescence. For example, miR-181a is upregulated during the senescence of human dermal fibroblasts which subsequently induces cellular senescence in early-passage cells.^16^ Furthermore, downregulated miR-181a was shown to attenuate oxidative stress in myocardial injury by interacting with XIAP.^17^ Nevertheless, the potential mechanism of miR-181a in H_2_O_2_-induced cellular senescence is unclear.

Protein disulfide isomerase family A member 6 (PDIA6, also known as P5) catalyzes protein folding and displays isomerase and chaperone activities. ^18^ Intriguingly, a previous study demonstrated that PDIA6 is downregulated during cellular senescence in BMSCs.^19^ In addition, PDIA6, localized in mitochondria, was shown to inhibit cell death caused by oxidative stress.^20^ However, whether PDIA6 exerts an effect on H_2_O_2_-induced cellular senescence in human foreskin fibroblasts (HFF) is unknown.

In this study, the authors intended to explore the role and mechanism of miR-181a underlying H_2_O_2_-induced oxidative stress and cellular senescence in HFF. The results might help to develop a new perspective for ameliorating skin aging.

## Materials and methods

### Cell culture

HFF obtained from the cell bank of the chinese academy of sciences (Shanghai, China) were incubated in Dulbecco's modified Eagle’s medium (DMEM, Invitrogen, Carlsbad, CA, USA) containing 10% fetal bovine serum (Gibco, Grand Island, NY, USA) and 1% sodium pyruvate (Invitrogen) in a humidified incubator with at 37 °C with 5% CO_2_. After being grown to 80% confluence, cells in the logarithmic phase of growth were inoculated into 96-well plates (10^4^ cells/well) and further cultured for 24 h. Then, the culture medium was removed and replaced with DMEM containing 1% FBS. After 24 h of incubation, 0 or 200 μM H_2_O_2_ was added to the medium and maintained for 6 h to establish control HFF or H_2_O_2_-induced HFF. All experiments were performed in triplicate.

### Cell transfection

MiR-181a inhibitor and miR-NC were purchased from GenePharma (Shanghai, China) and were transfected into HFF (50 nM) for inhibiting miR-181a. Short hairpin RNA specifically targeting PDIA6 (sh-PDIA6), and control sh-NC also purchased from GenePharma were transfected into HFF (2 μg/μL) for downregulating PDIA6. Cell transfection was achieved by lipofectamine 2000 (Invitrogen) following the manufacturer’s recommendations. After 48 h, the transfection efficiency was assessed by RT-qPCR. All experiments were performed in triplicate.

### Cell counting Kit-8 (CCK-8) assay

After H_2_O_2_ treatment and/or indicated transfection, 10 μL of CCK-8 solution (Dojindo, Kumamoto, Japan) was added to the medium, and HFF were cultured at 37 °C for another 2 h. Then, a microplate reader (Molecular Devices, Shanghai, China) was utilized for measuring the absorbance at 450 nm according to the manufacturer’s instructions. All experiments were performed thrice.

### Reverse transcription-quantitative polymerase chain reaction (RT-qPCR)

Total RNA was isolated from HFF using TRIzol reagent (Invitrogen). Synthesis of cDNA was achieved by reverse transcription of total RNA using PrimeScript™ RT reagent Kit (Takara, Dalian, China). RT-qPCR was implemented using SYBR® Premix Ex Taq™ II (Takara) on a CFX96™ Real-Time System (Bio-Rad, Hercules, CA, USA). The relative expression levels of miR-181a and its downstream targets were calculated with the 2^−ΔΔCt^ method, with U6 and GAPDH as normalization, respectively. All experiments were performed in triplicate. Primer sequences are listed in Table 1.

**Table 1:** Primer sequences used for RT-qPCR.

Gene	Sequence (5’→ 3’)
hsa-miR-181a-5p forward	ACACTCCAGCTGGGAACATTCAACGCTGTCGG
hsa-miR-181a-5p reverse	TGGTGTCGTGGAGTCGA
PDIA6 forward	TCCTGCCCACTCCCTATCAA
PDIA6 reverse	GAACTGTATCCTCCGCTCCG
TNPO1 forward	GACGCGCCTACGGGA
TNPO1 reverse	TGTTGCACGGTTCTCTGGA
HMGB2 forward	GCCAACAGGCTCAAAGAA
HMGB2 reverse	CACACATTCCACACGCA
CBX4 forward	TGGAGTATCTGGTGAAATGGA
CBX4 reverse	ACGACGGGCAAAGGTAGGCAC
GAPDH forward	TGCACCACCAACTGCTTAGC
GAPDH reverse	GGCATGGACTGTGGTCATGAG
U6 forward	CTCGCTTGGGCAGCACA
U6 reverse	AACGCTTCACGAATTTGCGT

### Western blotting

HFF were lysed in RIPA buffer (Cell Signaling, Danvers, MA, USA) and the concentration of proteins was measured by a BCA kit (Bio-Rad). Protein samples (20 μg) were separated by 10% SDS-PAGE gels, transferred to polyvinylidene fluoride (PVDF) membranes (Millipore, Billerica, MA, USA), and blocked with 5% non-fat milk. Afterwards, the membranes were incubated at 4 °C overnight with primary antibodies as follows: anti-p21 (ab109520, 1:1000), anti-p53 (ab32389, 1:10000), anti-SMP30 (ab233007, 1:400), anti-beta-actin (ab115777, 1:200), anti-PDIA6 (ab154820, 1:1000) (all from Abcam, Cambridge, MA, USA) followed by incubation with secondary antibody (ab97080, Abcam) for 1 h at room temperature. Beta-actin was used as a loading control. The proteins were visualized with an ECL system (Bio-Rad) and quantified by ImageJ software (National Institutes of Health, Bethesda, MD, USA). All experiments were performed in triplicate.

### SA-β-gal staining

HFF were washed with PBS and fixed in 3% formaldehyde at room temperature for 15 min. After washing with PBS twice, HFF were incubated overnight at 37 °C with β-galactosidase staining reagents (1 mg/mL X-gal, 2 mM MgCl_2_, 150 mM NaCl, pH 6.0, 5 mM potassium ferrocyanide, 5 mM potassium ferricyanide and 40 nM citric acid/sodium phosphate: Beyotime, Shanghai, China). Eventually, stained HFF were imaged with a light microscope (Nikon, Tokyo, Japan) at 50× magnification, and the percentage of positive cells were analyzed with Image-Pro Plus 6.0 (Media Cybernetics, Silver Spring, MD, USA). All experiments were performed three times.

### Detection of SOD, GPx and CAT activities

The activities of SOD, GPx and CAT were measured as previously described.^21^ Briefly, one unit of SOD was defined as the amount of enzyme that suppressed 50% of the formazan/min. Xanthine and xanthine oxidase were utilized to produce superoxide anions. The reaction between superoxide anions and tetrasodium chloride formed yellow formazan whose absorption was evaluated at 450 nm. For GPx, enzymatic reactions in tubes containing reduced glutathione, glutathione reductase and NADPH, were initiated by adding cumene hydroperoxide. GPx activity was measured with a wavelength of 340 nm. For CAT activity, one unit of CAT was defined as the amount of enzyme that decomposed 1 M of H_2_O_2_/min. The rate of decomposition of H_2_O_2_ was assessed at 570 nm. The SOD, GPx and CAT assay kits (Jiancheng Bioengineering Institute, Nanjing, China) were used for spectrophotometrically detecting the enzyme activities which were expressed as U/mg of protein. All experiments were performed in triplicate.

### Luciferase reporter assay

The complementary binding site of miR-181a on PDIA6 3’UTR was predicted by TargetScan (http://www.targetscan.org/vert_71/). The wild-type or mutant sequences of PDIA6 3’UTR were subcloned into pmirGLO vectors (Promega, Madison, WI, USA) to establishPDIA6-Wt/Mut. Afterward, these vectors were co-transfected into HFF with miR-181a inhibitor or miR-NC using Lipofectamine 2000 (Invitrogen). After 48 h of transfection, the luciferase activity was evaluated by a dual-luciferase reporter kit (Promega), normalized to Renilla luciferase activity. All experiments were performed in triplicate.

### Statistical analysis

SPSS 18.0 (SPSS, Chicago, IL, USA) was utilized for statistical analysis. Data are presented as the mean ± standard deviation. Differences between two groups were analyzed by Student’s *t*-test while those among more than two groups were assessed by analysis of variance (ANOVA) followed by Tukey’s *post-hoc* analysis. Each experiment was repeated at least three times. The value of p < 0.05 was considered significant.

## Results

### Downregulation of miR-181a mitigates H_2_O_2_-induced cellular senescence and oxidative stress

**Figure 1:**
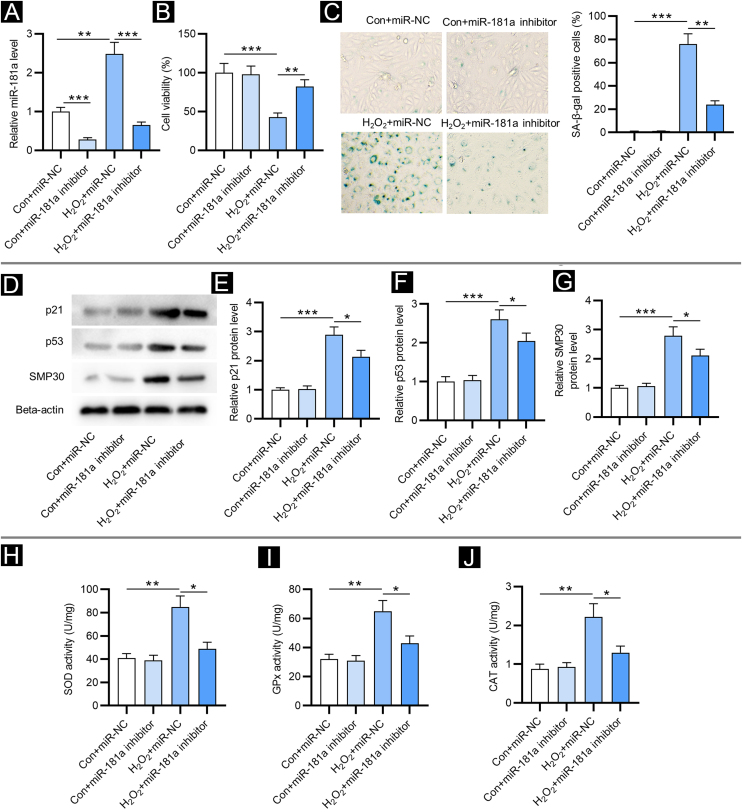
**MiR-181a depletion mitigates H_2_O_2_-induced cellular senescence and oxidative stress.** Assays were conducted in HFF with different treatments (with or without H_2_O_2_; transfection of miR-NC or miR-181a inhibitor). (A) RT-qPCR analysis of miR-181a level. (B) CCK-8 assay for evaluating cell viability. (C) SA-β-gal staining for assessing SA-β-gal activity. Cell pictures were imaged at 50× magnification. (D‒G) Western blotting for measuring protein levels of senescence markers. (H‒J) Measurement of the activities of SOD, GPx and CAT in HFF. *p < 0.05, **p < 0.01, ***p < 0.001.

First, the authors detected miR-181a levels in HFF by RT-qPCR. Relative to the control group, miR-181a level was increased in H_2_O_2_-treated HFF and was reduced after transfection of miR-181a inhibitor (Fig. 1A). Next, the impact of miR-181a on the viability of HFF was assessed. As revealed by CCK-8 assay, H_2_O_2_ treatment markedly reduced the viability of HFF, while miR-181a depletion attenuated this effect (Fig. 1B), suggesting that downregulated miR-181a might protect HFF against H_2_O_2_-induced cell damage. Moreover, detection of SA-β-gal activity displayed that miR-181a inhibition led to a reduction in the H_2_O_2_-induced increased percentage of SA-β-gal positive cells (Fig. 1C). A similar trend was observed in the results of western blotting. Protein levels of senescence markers (p21, p53 and SMP30) were significantly raised by H_2_O_2_ treatment in HFF compared to the control groups and were then decreased by miR-181a inhibitor (Fig. 1D‒G). The above results indicated that downregulated miR-181a alleviates H_2_O_2_-induced cellular senescence in HFF. Subsequently, the authors tested whether miR-181a had an impact on H_2_O_2_-induced oxidative stress. As shown by Figure 1 H‒J, H_2_O_2_ treatment markedly elevated the activity of SOD, GPx, and CAT, which was partially reversed after the downregulation of miR-181a in HFF. The results suggested that the antioxidant enzymes actively respond to oxidative stress and miR-181a inhibition can relieve H_2_O_2_-induced oxidative stress.

### MiR-181a targets PDIA6

**Figure 2:**
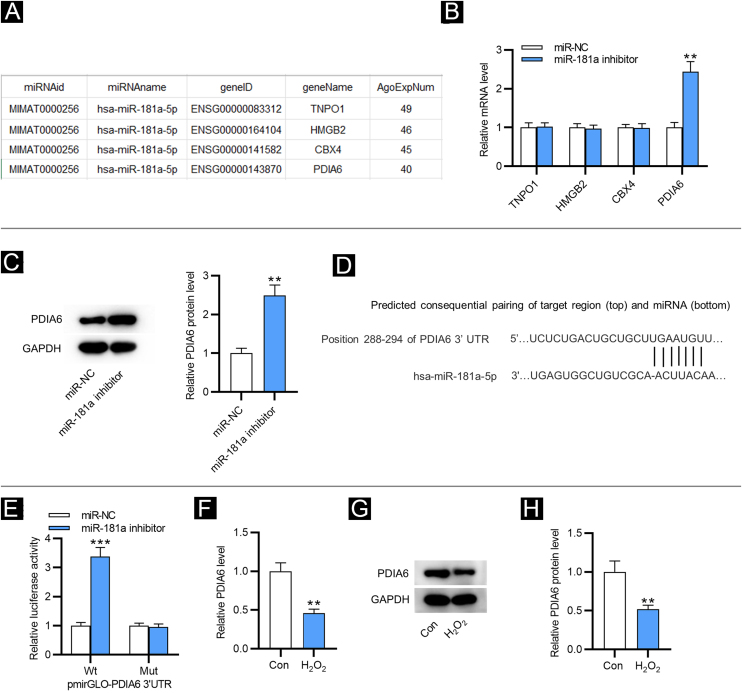
**MiR-181a binds with PDIA6.** (A) Four downstream targets of miR-181a predicted by ENCORI. (B) RT-qPCR analysis for assessing these mRNA levels in HFF transfected with miR-181a inhibitor. (C) Western blotting of PDIA6 protein expression in miR-181a inhibitor-transfected HFF. (D) The binding site of miR-181a on PDIA6 3’UTR predicted by TargetScan. (E) Luciferase reporter assay for elucidating the binding relation between PDIA6 and miR-181a. (F) RT-qPCR analysis of PDIA6 level in H_2_O_2_-treated HFF. G–H. Western blotting of PDIA6 protein level in H_2_O_2_-treated HFF and control group. **p < 0.01, ***p < 0.001.

As shown in Figure 2A, four downstream targets of miR-181a were screened out by ENCORI (https://starbase.sysu.edu.cn/) with the condition of a number of supported AGO CLIP-seq experiments (AgoExpNum) ≥40. Results from RT-qPCR showed that after inhibiting miR-181a in HFF, only the PDIA6 level was significantly enhanced (Fig. 2B). Likewise, western blotting displayed that miR-181a inhibitor increased the protein level of PDIA6 in HFF (Fig. 2C). The existence of putative complementary site between miR-181a and PDIA6 was predicted by TargetScan (Fig. 2D). Moreover, the luciferase activity of PDIA6-Wt was upregulated in HFF after transfection of miR-181a inhibitor, while that of PDIA6-Mut was not significantly influenced, as shown by luciferase reporter assay (Fig. 2E). Notably, RT-qPCR and western blotting demonstrated that mRNA and protein expression of PDIA6 was markedly downregulated in H_2_O_2_-treated HFF compared with the control group (Fig. 2F‒H). Collectively, PDIA6 is a target for miR-181a in HFF.

### PDIA6 knockdown reverses miR-181a inhibition-mediated suppressive impact on cellular senescence and oxidative stress

**Figure 3:**
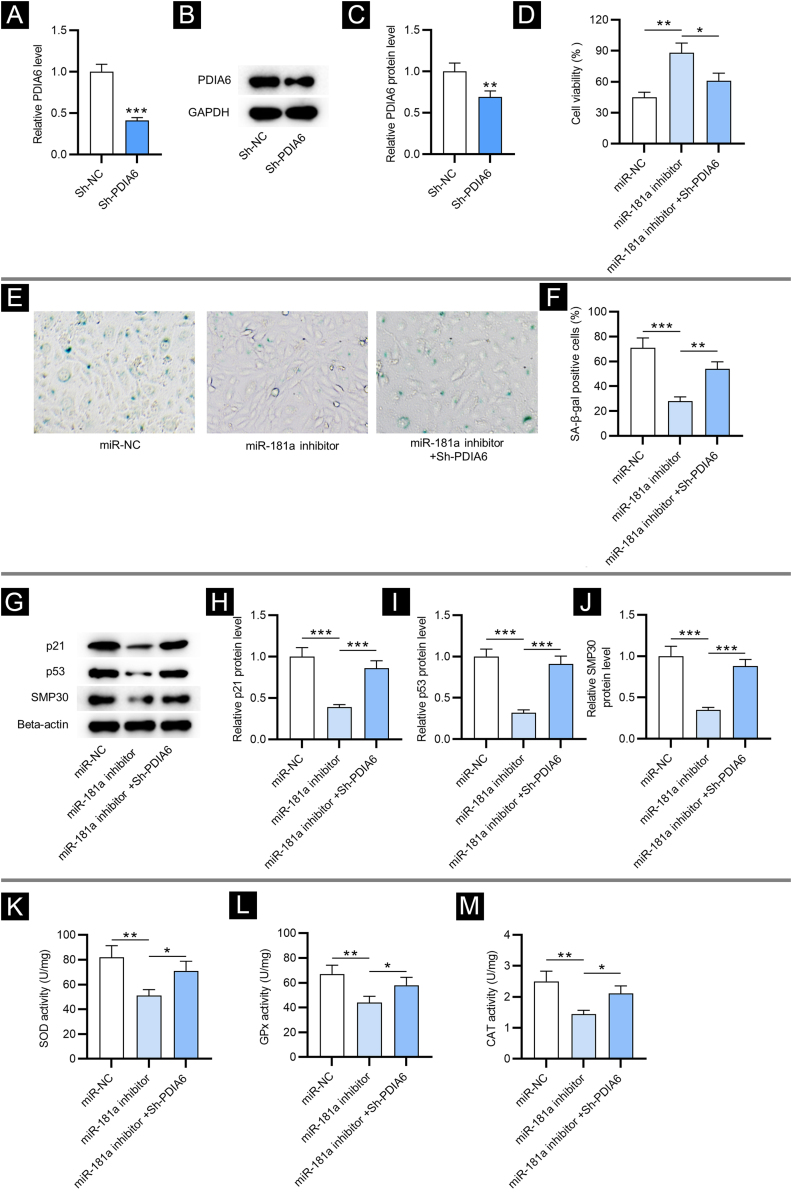
**PDIA6 knockdown reverses miR-181a inhibitor-mediated suppressive impact on cellular senescence and oxidative stress.** (A) RT-qPCR analysis of PDIA6 expression in sh-PDIA6-transfected HFF. (B‒C) Western blotting of PDIA6 protein expression in transfected HFF. (D) CCK-8 assay for evaluating the viability of HFF with transfection of miR-181a inhibitor, miR-181a inhibitor + sh-PDIA6 or miR-NC. (E‒F) SA-β-gal staining for assessing the percentage of SA-β-gal positive cells of HFF with above transfection. Cell pictures were imaged at 50× magnification. (G‒J) Western blotting for detecting concentration of senescence markers in HFF with above transfection. (K‒M) Measurement of the activities of SOD, GPx and CAT in HFF transfected with miR-181a inhibitor, miR-181a inhibitor + sh-PDIA6 or miR-NC. *p < 0.05, **p < 0.01, ***p < 0.001.

To investigate the impact of PDIA6 on cellular senescence in HFF, the authors first transfected sh-PDIA6 into HFF. The mRNA and protein expression levels of PDIA6 were decreased in HFF transfected with sh-PDIA6, as displayed by RT-qPCR and western blotting, respectively (Fig. 3A‒C). CCK-8 assay revealed that miR-181a inhibitor promoted the viability of HFF while co-transfection of sh-PDIA6 attenuated this effect (Fig. 3D). Additionally, results from SA-β-gal staining displayed that depletion of miR-181a significantly reduced the percentage of SA-β-gal positive cells, which was partially reversed by knocking down miR-181a and PDIA6 simultaneously (Fig. 3E‒F). This was consistent with western blotting which showed that knocking down PDIA6 rescued the reduction in senescence marker protein levels caused by miR-181a inhibitor in HFF (Fig. 3G‒J). Hence, it was suggested by the above results that downregulated miR-181a alleviates H_2_O_2_-mediated cellular senescence by targeting PDIA6.

Then, the authors tested whether miR-181a exerted its influence on oxidative stress by regulating PDIA6. As exhibited by the results, knocking down PDIA6 alleviated the suppressive effect on SOD, GPx and CAT activities in HFF resulting from miR-181a inhibitor (Fig. 3K‒M). This revealed that downregulated miR-181a alleviates H_2_O_2_-induced oxidative stress by targeting PDIA6.

## Discussion

The damage resulting from decreased antioxidant ability and imbalance of the oxidative system is termed oxidative stress.^22^ Emerging evidence has suggested that oxidative stress in the skin is a key cause of cellular senescence, consequently leading to skin aging.^23^, ^24^ Previous studies have elucidated that aging and ultraviolet B (UVB) exposure are strongly correlated with the high risk of skin cancer.^25^, ^26^ It is suggested that an increased presence of senescent fibroblasts in geriatric skin causes the silencing of insulin-like growth factor 1 (IGF-1) expression in the skin.^27^ The silencing of IGF-1 in senescent fibroblasts results in an inappropriate UVB-response and the proliferation of keratinocytes containing DNA damage, which ultimately leads to photocarcinogenesis.^26^ Hence, finding an effective approach to alleviate cellular senescence might help to reduce skin carcinogenic risk.

Numerous studies have demonstrated the significant effects of miRNAs involved in oxidative stress and cellular senescence, such as miR-1445-5p, miR-570, and miR-93-5p.^28^, ^29^, ^30^ Previous studies have verified that miR-181a plays a crucial role in cellular senescence.^31^ MiR-181a was reported to exhibit elevated expression in keratinocytes during replicative senescence, suggesting that overexpressed miR-181a might play a promotive role in cellular senescence.^32^ In this study, the authors examined the role of miR-181a in H_2_O_2_-treated HFF. Senescent cells are characterized by cell growth arrest and abnormal gene expression.^33^ Thus, the role of miR-181a in H_2_O_2_-treated HFF was identified by detecting its influences on cell viability, SA-β-gal staining, and expression levels of senescence markers. It was revealed that downregulated miR-181a could enhance cell viability and suppress cellular senescence caused by H_2_O_2_ in HFF. Furthermore, to detect the impact of miR-181a on H_2_O_2_-induced oxidative stress, the authors assessed the activities of the antioxidants (SOD, GPx and CAT) which are key regulators in the oxidative system. It was found that miR-181a inhibition significantly decreased the antioxidant abilities in H_2_O_2_-treated HFF, indicating that knocking down miR-181a is able to suppress oxidative stress induced by H_2_O_2_.

MiRNAs are recognized to modulate the expression of downstream targets by base-pairing to the sequences of mRNA 3’UTRs.^34^ To figure out how miR-181a exerts its influences on oxidative stress and cellular senescence, the bioinformatics tool ENCORI was utilized for screening the downstream targets of miR-181a. Among the selected mRNAs, the authors finally identified PDIA6 as the target gene of miR-181a. PDIA6, a member of the disulfide isomerase family, is implicated in various human diseases, such as diabetes mellitus, liver fibrosis, and various cancers.^35^, ^36^ Additionally, a previous study suggested that PDIA6 is closely related to cellular senescence in human BMSCs.^19^ In the present study, PDIA6 exhibited decreased expression in H_2_O_2_-treated HFF in comparison to the control group. Intriguingly, miR-181a inhibition alleviated H_2_O_2_- induced oxidative stress in HFF and cellular senescence, and knocking down PDIA6 reversed the inhibitory impact on cellular senescence and oxidative stress mediated by downregulated miR-181a. This suggested that silencing miR-181a mitigated H_2_O_2_-induced cellular senescence and oxidative stress by targeting PDIA6, and PDIA6 might protect HFF from cellular senescence induced by H_2_O_2_.

## Conclusion

In conclusion, the potential role and mechanism of miR-181a in regulating H_2_O_2_-induced oxidative stress and cellular senescence in HFF were investigated. The results revealed that downregulated miR-181a can ameliorate H_2_O_2_-induced oxidative stress and cellular senescence in HFF by regulating PDIA6. The present findings might help to develop a new perspective for improving skin aging and senescence-associated disorders.

## Financial support

The study was supported by the Medical Science Research Project of the Wuhan Municipal Health Commission (grant nº WX20Q03).

## Author's contribution

Yan Huang: Critical literature review; data collection; effective participation in research orientation; intellectual participation in propaedeutic and/or therapeutic management of studied cases; manuscript critical review; preparation and writing of the manuscript; statistical analysis; study conception and planning; approval of the final version of the manuscript.

Huimin Yan: Critical literature review; data collection; analysis and interpretation; effective participation in research orientation; intellectual participation in propaedeutic and/or therapeutic management of studied cases; manuscript critical review; preparation and writing of the manuscript; statistical analysis; study conception and planning; approval of the final version of the manuscript.

Yanqing Yang: Data collection; analysis and interpretation; approval of the final version of the manuscript.

Jinfei Zhou: Data collection; analysis and interpretation; approval of the final version of the manuscript.

Qijun Xu: Effective participation in research orientation; statistical analysis; approval of the final version of the manuscript.

Meng Hu: Critical literature review; data collection; analysis and interpretation; effective participation in research orientation; intellectual participation in propaedeutic and/or therapeutic management of studied cases; manuscript critical review; preparation and writing of the manuscript; statistical analysis; approval of the final version of the manuscript.

## Conflicts of interest

None declared.
